# Impact of hypertensive disorders of pregnancy on short- and long-term outcomes of pregnancy-associated hemorrhagic stroke

**DOI:** 10.3389/fneur.2023.1097183

**Published:** 2023-03-16

**Authors:** Mei Fang, Jiayan Wang, Zexu Wang, Yuqi Chen, Wei Xu, Chuanyuan Tao, Lu Ma, Chao You, Xin Hu, Fan Xia

**Affiliations:** ^1^Department of Neurosurgery, West China Hospital, Sichuan University, Chengdu, Sichuan, China; ^2^West China School of Medicine, Sichuan University, Chengdu, Sichuan, China

**Keywords:** hemorrhagic stroke, pregnancy, hypertensive disorders, outcome, preeclampsia, eclampsia

## Abstract

**Background and purpose:**

Though hypertension disorders of pregnancy (HDP) are recognized as independent pregnancy-associated stroke risk factors, few studies have considered their impact on stroke prognosis. Therefore, we intended to evaluate the impact of HDP on short- and long-term outcomes of pregnancy-associated hemorrhagic stroke (HS).

**Methods:**

We conducted a retrospective analysis of patients admitted to our hospital from May 2009 to December 2021 with a diagnosis of pregnancy-associated HS. After dividing patients into two groups by the presence of a diagnosis of HDP or not, the short- (at the time of discharge) and long-term (after discharge follow-up) outcomes were compared by mRS (modified Rankin Scale) scores, and poor functional outcome defined as mRS > 2. Adjusted odds ratios (OR) and 95% confidence intervals (CI) were reported.

**Results:**

Twenty-two HDP and 72 non-HDP pregnancy-associated HS patients were enrolled and follow-up after 4.7 ± 3.6 years. There was no significant difference between the two groups regarding short-term outcomes, but patients with HDP were more likely to reach poor functional outcomes at long-term follow-up (aOR = 4.47, 95% CI = 1.28–15.67, *p* = 0.019).

**Conclusions:**

In this retrospective study, women with hypertension disorders of pregnancy did not show worse short-term outcomes of pregnancy-associated hemorrhagic stroke compared to those without but had poorer long-term functional outcomes. This underlines the importance of prevention, recognition, and treatment of hypertension disorders in these women.

## 1. Introduction

Pregnancy-associated stroke (PAS) is defined as a stroke occurring during pregnancy, delivery, or within 6 weeks postpartum. It is a rare but catastrophic pregnancy complication that may seriously affect maternal and infant outcomes. Unlike strokes in the general population, which are dominated by ischemic, hemorrhagic stroke (HS) accounts for roughly half of PAS (1, 2). According to previous studies, HS is more highly associated with maternal mortality compared to other types of strokes (3–5).

Hypertensive disorders of pregnancy (HDP) are recognized risk factors of pregnancy-associated HS, affecting 7.3% of all pregnancies (6–9). These disorders consist of the following five categories: (1) chronic hypertension, defined as high blood pressure (BP) predating the pregnancy or discovered before 20 weeks gestation; (2) gestational hypertension, refers to persistent new onset hypertension that develops at or after 20 weeks gestation; (3) pre-eclampsia, refers to diagnosed hypertension between 20 weeks of gestation and puerperium, accompanied by proteinuria or multisystem organ failure; (4) eclampsia, refers to convulsions occur during pregnancy and puerperium, and cannot be attributed to any other pre-existing neurological disease; (5) pre-eclampsia superimposed upon chronic hypertension (9). In addition, there is also a HELLP (H = Hemolysis, EL = Elevated Liver enzymes, LP = Low Platelets) syndrome, which is currently regarded as a variant of severe preeclampsia in some degree (10). Among all types of PAS, HS has the greatest correlation with HDP (11). High-capacity circulation, endothelial dysfunction, and blood-brain barrier disruption resulting from HDP may play a key role in increasing the risk of developing HS during pregnancy and puerperium (6, 12).

Despite HDP having been confirmed as a strong risk factor for stroke in women of childbearing age, there is a dearth of literature examining its impact on PAS outcome, as the majority have conducted prognosis studies in the wider pregnant population (1, 11, 13). Therefore, in the present study, we intended to evaluate the impact of HDP on short-term and long-term outcomes of pregnancy-associated HS to promote a better understanding of their relevance. To our best knowledge, this is the first study considering the long-term functional outcome of PAS.

## 2. Method

The study was approved by the Ethics committee of West China Hospital of Sichuan University, Chengdu, China, and conducted according to the Declaration of Helsinki. Written informed consent was obtained from each patient or legal surrogate.

### 2.1. Patient selection

From May 2009 to December 2021, we retrospectively reviewed all patients who suffered from HS during pregnancy or the first 6 weeks of postpartum in West China Hospital, a tertiary hospital. HS was defined when there was a rapid development of focal neurological deficit caused by a rupture of a blood vessel, and it can be further subdivided into intracerebral hemorrhage (ICH) and subarachnoid hemorrhage (SAH). The selection criteria were as follows.

#### 2.1.1. Inclusion criteria

(1) ICH or SAH was confirmed by cranial computed tomography, magnetic resonance imaging, or angiography. In patients with highly suspected SAH with negative CT, a diagnosis of SAH could also be made through a lumbar puncture.

(2) Pregnancy information can be obtained, and patients confirmed to be within the pregnancy, delivery, or puerperium.

#### 2.1.2. Exclusion criteria

(1) ICH or SAH caused by trauma.
(2) ICH or SAH secondary to malignant tumor stroke.
(3) Presented as hemorrhage transformation after ischemic stroke.
(4) Intracranial bleeding in the chronic stage.
(5) Ectopic pregnancy was confirmed by ultrasonography.

### 2.2. Data collection

The following data were collected: age at stroke onset, previous stroke history, chronic diseases, gestation comorbidities, BP at admission, consciousness state at admission, developed stroke type (ICH or SAH or mixed), stroke onset time (antepartum/intrapartum/postpartum), initial symptoms, underlying cerebral vascular structural changes, length of stay, disposition place, stroke-related complications, treatment, modified Rankin Scale (mRS) scores at discharge and last follow-up. The long-term functional outcomes were obtained through telephone inquiries or outpatient visits, and the last follow-up was conducted in May 2022.

### 2.3. Outcome measured

After including all eligible patients, we would divide the patients into two groups based on the presence of a diagnosis of HDP or not. The functional outcomes were assessed by the modified Rankin Scale (mRS), which measures the degree of disability or dependence in people's daily activities. The main hypothesis we tested was that in pregnancy-associated HS women, those with HDP are more likely to reach poor functional outcomes (score 3–6 on the mRS) at discharge and even in the future when compared to those without HDP. Therefore, the primary outcomes for this analysis were the comparison of short-term (at the time of discharge) and long-term (after discharge follow-up) functional outcomes between the two groups. The mRS scores were independently assessed by two trained researchers, and if there were disagreements, a consensus was reached after discussion. The pre-specified secondary outcomes included: (1) in-hospital and long-term mortality, discharge location, and length of hospital stay; (2) incidence of stroke-related complications (14) (brain hernia, intracranial infection, secondary epilepsy, pulmonary and urinary tract infection, cardiac complication (including heart failure and myocardial injury), acute upper gastrointestinal bleeding) and the necessity for life support therapy (endotracheal intubation, tracheotomy, blood transfusion, intravenous nutrition, vasoactive drug therapy).

### 2.4. Statistical analysis

Descriptive statistics were addressed. We reported means with standard deviations for quantitative variables and numbers with their frequency (%) for qualitative variables. Comparison of ranked variables or continuous variables with non-normal distributions was analyzed by the Mann–Whitney *U* test, and continuous variables with normal distribution were analyzed by *t*-test. Pearson's chi-squared tests were performed to compare categorical variables. Then, we implemented multivariable logistic regression analysis after adjusting for age and admission consciousness level to investigate the effect of HDP on the prognosis of pregnancy-associated HS. Length of hospital stay was partitioned according to the median level (10 days) of total patients, and prolonged hospital stay was defined as ≥10 days. Odds ratios (ORs) with 95% confidence intervals (CIs) were reported.

All data were analyzed using SPSS, version 26.0 (IBM, New York, NY), and a two-sided *P* < 0.05 was considered as statistical significance.

## 3. Results

Ninety-four pregnancy-associated HS patients were enrolled, of whom twenty-two were with diagnosed HDP during recent pregnancy. Table 1 summarizes the demographic information and stroke-associated clinical condition. The young women included in this study had almost no chronic diseases other than hypertension (only one nephrotic syndrome in the HDP group and one congenital heart disease in the non-HDP group). Therefore, in the following data analysis process, we neglected the presence of comorbidity. Headache [11 (50%) vs. 49 (68.1%)] was the most common initial symptom in both groups and there was no significant difference in the initial symptom between the two groups except disturbance of consciousness (45.5 vs. 19.4%, *p* = 0.014). In addition, patients with HS with HDP tended to present worse consciousness levels when admitted (*p* = 0.003). Table 2 summarizes the general conditions of patients in the HDP group. There were five chronic hypertension, five gestational hypertension, four pre-eclampsia, five eclampsia, and three pre-eclampsia superimposed upon chronic hypertension. Among five patients with eclampsia, two had developed HELLP syndrome, and three of the whole patients (3/22, 13.6%) indicated that they had also suffered from HDP in the previous pregnancy.

**Table 1:** Baseline characteristics of patients.

	**ALL (*n* = 94)**	**HDP patients (*n* = 22)**	**Non-HDP patients (*n* = 72)**	***P*-value**
**Age at stroke onset**	29.7 ± 6.4	31.8 ± 5.1	29.0 ± 6.6	0.077
**Blood pressure at admission**				
Systolic blood pressure	129.7 ± 28.5	159.1 ± 32.2	120.7 ± 20.1	< 0.001^*^
Diastolic blood pressure	81.4 ± 16.5	97.6 ± 16.6	76.4 ± 13.0	< 0.001^*^
**Hemorrhagic stroke type**				0.864
Intracranial hemorrhage	67 (71.3%)	16 (72.7%)	51 (70.8%)	
Subarachnoid hemorrhage	27 (28.7%)	6 (27.3%)	21 (29.2%)	
**Initial symptoms**				
Headache	60 (63.8%)	11 (50.0%)	49 (68.1%)	0.123
Vomiting	37 (39.4%)	7 (31.8%)	30 (41.7%)	0.408
Disturbance of consciousness	24 (23.4%)	10 (45.5%)	14 (19.4%)	0.014^*^
Nausea	21 (22.3%)	4 (18.2%)	17 (23.6%)	0.808
Weakness/paresis	18 (19.1%)	3 (13.6%)	15 (20.8%)	0.659
Dizziness	14 (14.9%)	3 (13.6%)	11 (15.3%)	1.000
Convulsion	11 (11.7%)	4 (18.2%)	7 (9.7%)	0.483
Aphasia/language disturbance	6 (6.4%)	1 (4.5%)	5 (6.9%)	1.000
Visual impairment	4 (4.3%)	2 (9.1%)	2 (2.8%)	0.496
**Level of consciousness**				0.003^*^
Awake	44 (46.8%)	4 (18.2%)	40 (55.6%)	
Lethargy or obtundation	26 (27.7%)	7 (31.8%)	19 (26.4%)	
Coma	24 (25.5%)	11 (50.0%)	13 (18.1%)	
**Previous stroke history**	4 (4.3%)	0 (0%)	4 (5.6%)	0.591
**Vascular abnormalities**				< 0.001^*^
Arteriovenous malformations	23 (24.5%)	0 (0%)	23 (31.9%)	
Aneurysm	17 (18.1%)	3 (13.6%)	14 (19.4%)	
Cavernous hemangioma	6 (6.4%)	0 (0%)	6 (8.3%)	
Cerebral venous thrombosis	8 (8.5%)	2 (9.1%)	6 (8.3%)	
Moyamoya disease	3 (3.2%)	0 (0%)	3 (4.2%)	
Mixed	3 (3.2%)	0 (0%)	3 (4.2%)	
None	34 (36.2%)	17 (77.3%)	17 (23.6%)	
**Stroke onset time**				0.733
Before delivery	64 (68.1%)	16 (72.2%)	48 (66.7%)	
1^st^ trimester	6 (6.4%)	3 (13.6%)	3 (4.2%)	
2^nd^ trimester	19 (20.2%)	1 (4.5%)	18 (25.0%)	
3^rd^ trimester	39 (41.5%)	12 (54.5%)	27 (37.5%)	
During delivery	3 (3.2%)	1 (4.5%)	2 (2.8%)	
After delivery	27 (28.7%)	5 (22.7%)	22 (30.6%)	
**Treatment**				0.344
Conservative	43 (45.7%)	12 (54.5%)	31 (43.1%)	
Surgery	51 (54.3%)	10 (45.5%)	41 (56.9%)	
Hematoma evacuation	13 (14.0%)	4 (18.2%)	8 (12.5%)	
External ventricular drains	10 (10.8%)	2 (9.1%)	6 (8.3%)	
Aneurysm clipping	9 (9.7%)	4 (18.2%)	5 (7.0%)	
Microsurgical resection	16 (17.2%)	–	16 (22.5%)	
Endovascular treatment	5 (5.4%)	–	5 (7.0%)	
Decompressive craniectomy	2 (2.2%)	–	1 (1.4%)	
**Short time follow-up duration (days)**	14.2 ± 16.0	16.1 ± 15.7	13.6 ± 16.1	0.529
**Long time follow-up duration (years)**	4.7 ± 3.6	4.8 ± 4.0	4.6 ± 3.6	0.829

^*^P < 0.05.

HDP, hypertensive disorders of pregnancy.

**Table 2:** Brief description of all patients in hypertensive disorders (HDP) of pregnancy group.

**Number**	**HDP types**	**Bleeding type**	Onset time*	**Complications**	**Treatments**	**Poor outcome at discharge^†^**	**Poor outcome at follow-up^†^**
1	Pre-eclampsia + chronic hypertension	ICH	3^rd^	Pulmonary infection, AUGIB	Hematoma evacuation	1	1
2	Pre-eclampsia + chronic hypertension	ICH	Postpartum	Pulmonary infection, AUGIB, secondary epilepsy	Conservative	0	0
3	Gestational hypertension	SAH	Postpartum	Pulmonary infection	Aneurysm clipping	1	1
4	Gestational hypertension	SAH	3^rd^		Aneurysm clipping	1	0
5	Chronic hypertension	SAH	3^rd^	Cerebral herniation	Conservative	1	1
6	Pre-eclampsia	ICH	3^rd^	Cardiac dysfunction	Conservative	0	1
7	Eclampsia	ICH	3^rd^	Cerebral herniation, cardiac dysfunction	Conservative	1	1
8	Eclampsia	ICH	3^rd^	Pulmonary infection, urinary infection, secondary epilepsy, cardiac dysfunction	Conservative	1	1
9	Gestational hypertension	SAH	Postpartum		Aneurysm clipping	0	1
10	Eclampsia	ICH	3^rd^	Urinary infection	Conservative	1	1
11	Chronic hypertension	ICH	3^rd^	Cerebral herniation, cardiac dysfunction	External ventricular drains	1	1
12	Pre-eclampsia	ICH	Postpartum		Conservative	1	1
13	Eclampsia	ICH	Intrapartum		Conservative	0	0
14	Chronic hypertension	ICH	Postpartum		Conservative	1	Missing
15	Pre-eclampsia	ICH	3^rd^	Pulmonary infection, cerebral herniation	Hematoma evacuation	1	1
16	Chronic hypertension	ICH	1^st^		External ventricular drains	1	Missing
17	Eclampsia	SAH	3^rd^	Urinary infection	Conservative	1	0
18	Pre-eclampsia	ICH	3^rd^	Intracranial infection, Pulmonary infection	Hematoma evacuation	1	1
19	Pre-eclampsia + chronic hypertension	ICH	3^rd^	Secondary epilepsy, cardiac dysfunction	Conservative	0	1
20	Chronic hypertension	SAH	1^st^	Cardiac dysfunction	Aneurysm clipping	1	0
21	Gestational hypertension	ICH	1^st^		Conservative	1	0
22	Gestational hypertension	ICH	Intrapartum	Intracranial infection, urinary infection, cardiac dysfunction	Hematoma evacuation + external ventricular drains	1	1

^*^Patients suffered stroke during pregnancy were indicated by their trimester.

^†^Poor outcome refers to score 3–6 on the mRS scores, and 1 indicates poor outcome, 0 indicates the opposite.

AUGIB, acute upper gastrointestinal bleeding.

**Figure 1:**
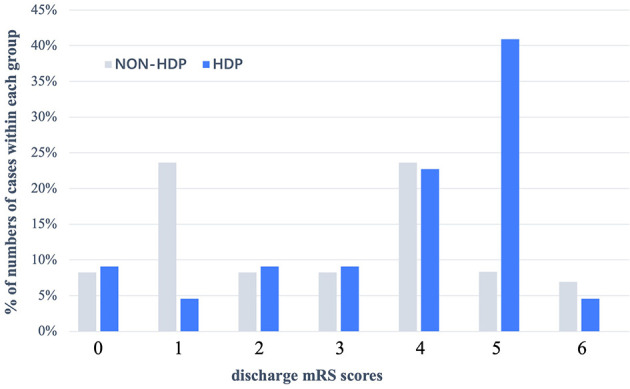
Distribution (expressed as a percentage) of mRS scores at discharge in patients with HDP and without HDP in pregnancy—associated hemorrhagic stroke. In patients without HDP, their discharge mRS score were evenly distributed, but in patients with HDP, discharge with mRS = 4 (22.7%) or mRS = 5 (40.9%) accounted for the main proportion. HDP, hypertensive disorder of pregnancy; mRS scores, modified Rankin Scale.

Table 3 summarizes the short-term outcomes of included patients. Despite patients diagnosed with HDP during recent pregnancy showed poorer functional outcomes at discharge in univariate analysis (OR = 3.22, 95% CI = 1.07–9.65, *p* = 0.037), there was no significant difference between the two groups after adjustment (77.3 vs. 51.4%, aOR = 1.43, 95% CI = 0.41–4.96, *p* = 0.572). Figure 1 summarizes the distribution of mRS scores within each group at discharge, from which we could directly figure out that mRS scores are concentrated at high scores in the HDP cohort but evenly distributed in the non-HDP cohort.

**Table 3:** Odds ratios (ORs) with confidence interval (CI)^*^ for short-term outcomes by hypertensive disorders of pregnancy (HDP) status among pregnancy-related hemorrhagic stroke.

**Short-term outcomes**	**Total number of events**		**OR with 95% CI** ^*^		***P*-value**
	**HDP group (*****n*** = **22)**	**Non-HDP (*****n*** = **72)**	**Unadjusted**	**Adjusted** ^†^	
In-hospital death	1 (4.5%)	5 (7.0%)	0.64 [0.07, 5.77]	0.36 [0.04, 3.59]	0.385
**mRS scores at discharge**					
mRS 3–6	17 (77.3%)	37 (51.4%)	3.22 [1.07, 9.65]^§^	1.43 [0.41, 4.96]	0.572
**Length of stay**			1.93 [0.71, 5.24]	1.20 [0.40, 3.57]	0.746
< 10	13 (59.1%)	53 (73.6%)			
≥10	9 (40.9%)	19 (26.4%)			
**Disposition place**			2.72 [0.91, 8.17]	2.01 [0.61, 6.57]	0.249
Home	5 (22.7%)	32 (44.4%)			
Non-routine^‡^	17 (77.3%)	40 (55.6%)			

^*^Referent group: hospitalizations with non-hypertensive disorders of pregnancy-associated hemorrhagic stroke.

^†^Adjusted for age and level of consciousness at admission.

^‡^Non-routine disposition: death, short-term hospital, and other facilities.

^§^p < 0.05.

mRS, modified Rankin Scale.

Table 4 summarizes the stroke-related clinical complication and life-support therapy received by patients during hospitalization. Except for univariate analysis on intravenous nutrition usage, there was no statistically significant difference in the incidence of stroke-related complications or life-support therapy between the two groups.

**Table 4:** Odds ratios (ORs) with confidence interval (CI)^*^ for complications and life-support therapy by hypertensive disorders of pregnancy (HDP) status among hemorrhagic stroke related to pregnancy.

**Complications and life-support therapy**	**Total number of events**		**OR with 95% CI** ^*^		***P*-value**
	**HDP group (*****n*** = **22)**	**Non-HDP (*****n*** = **72)**	**Unadjusted**	**Adjusted** ^†^	
**Complication**					
Cerebral herniation	4 (18.2%)	8 (11.1%)	1.78 [0.48, 6.5 8]	0.73 [0.17, 3.16]	0.669
Intracranial infection	2 (9.1%)	2 (2.8%)	3.50 [0.46, 26.44]	2.31 [0.28, 19.33]	0.440
Secondary epilepsy	3 (13.6%)	10 (13.9%)	0.98 [0.24, 3.93]	1.08 [0.23, 5.21]	0.919
Pulmonary infection	6 (27.3%)	16 (22.2%)	1.31 [0.44, 3.91]	0.66 [0.19, 2.27]	0.513
Urinary tract infection	4 (18.2%)	8 (11.1%)	1.78 [0.48, 6.58]	3.29 [0.66, 16.39]	0.146
Cardiac complication^‡^	7 (31.8%)	13 (18.1%)	2.12 [0.72, 6.23]	1.44 [0.43, 4.89]	0.556
AUGIB	2 (9.1%)	1 (1.4%)	7.10 [0.61, 82.38]	4.94 [0.34, 71.86]	0.242
**Life-support therapy**					
Tracheotomy	4 (18.2%)	4 (5.6%)	3.78 [0.86, 16.60]	1.67 [0.33, 8.41]	0.531
Endotracheal intubation	13 (59.1%)	35 (48.6%)	1.71 [0.65, 4.50]	0.88 [0.29, 2.67]	0.826
Intravenous nutrition	11 (50.0%)	17 (23.6%)	3.24 [1.19, 8.77]^§^	2.07 [0.71, 6.07]	0.185
Blood transfusion	8 (36.4%)	13 (18.1%)	2.59 [0.90, 7.46]	1.73 [0.51, 5.84]	0.378
Vasoactive drugs	7 (31.8%)	12 (16.7%)	2.33 [0.78, 6.94]	1.33 [0.40, 4.36]	0.643

^*^Referent group: hospitalizations with non-hypertensive disorders of pregnancy-associated hemorrhagic stroke.

^†^Adjusted for age and level of consciousness at admission.

^‡^Cardiac complication includes heart failure and myocardial injury.

^§^p < 0.05.

AUGIB, acute upper gastrointestinal bleeding.

Table 5 summarizes the long-term outcomes among the two groups. Follow up averaged 4.7 ± 3.6 years, the total missing rate was 10.64% (10/94, two patients in the HDP group and eight patients in the non-HDP group), and there was still no significant difference in death rate between the two groups at the last follow-up (30.0 vs. 12.5%, aOR = 1.55, 95% CI = 0.40–5.92, *P* = 0.526). However, patients in HDP groups showed much poorer functional outcomes than those patients in non-HDP groups, and a significant difference was maintained even after adjusting for confounders (70.0 vs. 23.4%, aOR = 4.47, 95% CI = 1.28–15.67, *P* = 0.019).

**Table 5:** Odds ratios (ORs) with confidence interval (CI)^*^ for long-term outcomes by hypertensive disorders of pregnancy (HDP) status among pregnancy-related hemorrhagic stroke.

**Long-term outcomes**	**Total number of events** ^**‡**^		**OR with 95% CI** ^*^		***P*-value**
	**HDP group (*****n*** = **20)**	**Non-HDP (*****n*** = **64)**	**Unadjusted**	**Adjusted** ^†^	
Death at last follow up	6 (30.0%)	8 (12.5%)	3.00 [0.90, 10.06]	1.55 [0.40, 5.92]	0.526
**mRS scores at last follow up**					
mRS 3-6	14 (70.0%)	15 (23.4%)	7.62 [2.49, 23.31]^§^	4.47 [1.28, 15.67]^§^	0.019

^*^Referent group: hospitalizations with non-hypertensive disorders of pregnancy-associated hemorrhagic stroke.

^†^Adjusted for age, level of consciousness at admission.

^‡^Total missing rate was 10.64% (10/94, two in HDP group and eight in non-HDP group).

^§^p < 0.05.

mRS, modified Rankin Scale.

## 4. Discussion

Despite that a considerable part of stroke caused by HDP could be prevented through early identification and treatment, the incidence of stroke among women with HDP still increased over time (15, 16). Our study is the first to systematically evaluate the impact of HDP on pregnancy-associated HS prognosis. The main findings of this study are as follows: (1) Among pregnancy-associated HS patients, the long-term functional outcomes of patients diagnosed with HDP were significantly poorer; (2) HDP may not impact the short-term outcomes of pregnancy-associated HS neither increase the occurrence of stroke-related complications or usage for life-support therapy.

For PAS, HDP has always been the focus of attention. Previous studies have established that HDP is an important independent risk factor for HS in women of childbearing age (16–19). In addition, compared with other etiologies of HS such as aneurysms or arterial malformations, HDP possesses the valuable characteristic of “preventable” (4, 18, 20). Inadequate BP control is blamed as the most common mistake for stroke-related maternal death, in other words, adequate BP control may give maximum preventability to stroke-induced poor outcomes in these patients (21). As is known to all, elevated BP is the cornerstone of HDP, which means a considerable proportion of HDP-related stroke deaths can be avoided if timely and correctly treated. Oral labetalol, methyldopa, and nifedipine are the frequently recommended antihypertensive medications during pregnancy, and intravenous hydralazine should be additionally added for acute hypertensive urgency (22). However, despite all guidelines acknowledging the significance of BP control, there is still no consensus on the target BP level for pregnant women due to a lack of high-quality evidence. Further studies on lowering BP therapy among pregnant women with HS are warranted.

Another key element to treat HDP is “placenta delivery” (23). Though the pathophysiology of HDP has yet to be fully elucidated, the theory that the release of antiangiogenic factors caused by placental vascular maldevelopment leads to maternal endothelial dysfunction and resulting hypertension is generally accepted (24). According to the International Society for the Study of Hypertension in Pregnancy guidelines and the American College of Obstetricians and Gynecologists Task Force, in women with preeclampsia with severe features or eclampsia, emergency delivery soon after maternal stabilization is indicated irrespective of gestational age (9, 25). Although our study eliminated the obstetric complication analysis due to its high data missing rate, the roughly estimated preterm birth rate was extremely higher within the HDP group (40.9 vs. 16.9%).

It can be seen that most of our patients (both in the HDP and non-HDP groups) had their onset in the third trimester and puerperium, which is in line with the previous study's findings (11, 26). This may be related to the dramatic increase in plasma volume and cardiac output in the third trimester, which is attributed to the general vasodilatation of the cardiovascular system combined with an increase in water volume due to water retention (27, 28). While in the puerperium, all the above changing parameters rapidly return to normal, leading to an increase in vascular osmotic pressure, a decrease in blood volume, and a relatively vascular constriction, which eventually leads to a mild increase in blood pressure (28). This set of changes may noticeably increase the risk of postpartum intracranial bleedings.

There is limited literature on the clinical outcomes of pregnant women with HS, especially on long-term outcomes. In a study compared the short-term outcomes (in-hospital mortality and home-discharge rate) between pregnant and non-pregnant HS patients, they found that pregnant SAH patients had lower in-hospital mortality, higher home discharge and independent ambulation rate than their non-pregnant counterparts, and pregnant ICH patients also had lower odds of in-hospital mortality than non-pregnant ICH patients (13). The reported overall (ICH+SAH) in-hospital mortality of pregnant-associated HS in this study was 6.7%, which is similar to our results (6.4%). A previous study using the 1994–2011 Nationwide Inpatient Sample examined the stroke-related complication and showed that among stroke hospitalizations, patients with HDP had higher rates of complications than those without it (16). However, this study did not conduct further analysis based on stroke subtypes, and worse outcomes in the HDP group may be associated with a higher proportion of HS which generally induces more severe clinical conditions. Race is a proven independent risk factor for stroke occurrence whether in women of whole childbearing age or gravida with HDP, and it is also an influencing factor related to maternal outcomes of PAS (7, 29, 30). Some Asian population-based studies have shown that Asian women are prominently more likely to develop HS during pregnancy or puerperium compared to western countries (18, 19). Therefore, it is crucial to identify the impact of HDP on the prognosis of pregnancy-associated HS based on Asian women.

Consistent with the characteristics of “hypertensive” of HDP, the admission BP is significantly higher in the HDP group in our study. Some studies argued that the pathophysiology of stroke concerning HDP is similar to hypertensive encephalopathy in that the increase of BP results in the automatic regulation disturbance of cerebral blood flow, cerebral hyper-perfusion, blood-brain barrier disruption, brain edema, and hypertension-induced vessel wall damage (15, 31, 32). However, many women who develop stroke in the setting of preeclampsia or eclampsia were exactly at BP within the normal range of cerebral autoregulation which is considerably lower than those reported for hypertensive encephalopathy (33). Thus, elevated BP is not the only causative factor for this condition, and other mechanisms, such as endothelial dysfunction, increased sympathetic activity, and hyperreflexia that occurs in pre-eclampsia may also be involved (33–35). Further research on the pathophysiology of stroke related to HDP is warranted.

There are several limitations to be noted. First, this is a single-center retrospective study. The number of included patients is small, which restricted us from performing subgroup analysis within each HDP subtype. In addition, some data may be missing due to its retrospective nature, such as maternal birth-related complications. Second, it was reported that subjects with anemia, heart disease, or gestational diabetes mellitus, which factors are also correlated with stroke outcomes, have a higher risk of HDP occurrence when compared to their corresponding controls (36, 37). However, we did not include these confounders in our multivariable logistic regression due to the limited sample size. Third, due to the retrospective design and the large confidence interval of follow-up, the collection of specific data regarding potential confounders such as rehabilitation activities, complications, and readmission status of patients after discharge was insufficient. Fourth, the number of fetuses and the time of pregnancies are associated with the stroke risk (38), and the possibility of their impact on outcomes cannot be ruled out. Due to the limited availability of relevant clinical data, our study omitted this part's analysis, which may bias the results. Our study for the first time determines the impact of HDP on short-term and long-term outcomes of pregnancy-associated HS by systematically comparing in-hospital death, discharge, and post-hospital mRS score, stroke-related complications, and life-support therapies. We hope this study could lay the groundwork for future research on this topic.

## 5. Conclusions

In this retrospective study, women with hypertension disorders of pregnancy did not show worse short-term outcomes of pregnancy-associated hemorrhagic stroke compared to those without but had poorer long-term functional outcomes. This underlines the importance of prevention, recognition, and treatment of hypertension disorders in these women.

## Data availability statement

The original contributions presented in the study are included in the article/supplementary material, further inquiries can be directed to the corresponding authors.

## Ethics statement

The studies involving human participants were reviewed and approved by Ethics Committee of West China Hospital of Sichuan University, Chengdu, China. The patients/participants provided their written informed consent to participate in this study.

## Author contributions

Conceptualization and writing—original draft preparation: MF. Validation: WX and CT. Investigation: JW and ZW. Writing—review and editing: FX. Visualization: MF, YC, and FX. Supervision: LM and CY. Project administration: XH. All authors approved the final version of the manuscript.
